# Challenges and New Approaches to Proving the Existence of Muscle Synergies of Neural Origin

**DOI:** 10.1371/journal.pcbi.1002434

**Published:** 2012-05-03

**Authors:** Jason J. Kutch, Francisco J. Valero-Cuevas

**Affiliations:** 1 Division of Biokinesiology & Physical Therapy, University of Southern California, Los Angeles, California, United States of America; 2 Department of Biomedical Engineering, University of Southern California, Los Angeles, California, United States of America; University College London, United Kingdom

## Abstract

Muscle coordination studies repeatedly show low-dimensionality of muscle activations for a wide variety of motor tasks. The basis vectors of this low-dimensional subspace, termed muscle synergies, are hypothesized to reflect neurally-established functional muscle groupings that simplify body control. However, the muscle synergy hypothesis has been notoriously difficult to prove or falsify. We use cadaveric experiments and computational models to perform a crucial thought experiment and develop an alternative explanation of how muscle synergies could be observed without the nervous system having controlled muscles in groups. We first show that the biomechanics of the limb constrains musculotendon length changes to a low-dimensional subspace across all possible movement directions. We then show that a modest assumption—that each muscle is independently instructed to resist length change—leads to the result that electromyographic (EMG) synergies will arise without the need to conclude that they are a product of neural coupling among muscles. Finally, we show that there are dimensionality-reducing constraints in the isometric production of force in a variety of directions, but that these constraints are more easily controlled for, suggesting new experimental directions. These counter-examples to current thinking clearly show how experimenters could adequately control for the constraints described here when designing experiments to test for muscle synergies—but, to the best of our knowledge, this has not yet been done.

## Author Summary

How the brain and spinal cord control the body is a fundamental question of critical scientific and clinical importance. The preferred experimental approach to answer this question has been to infer the neural control strategy by analyzing recordings of muscle activity and limb mechanics collected while animals and people use their limbs. This has led to a popular, but not yet proven, hypothesis that the brain and spinal cord simplify the control of the numerous muscles by grouping them into few functional units called neural synergies. Our detailed experiments and simulations challenge the utility of this approach and the validity of its interpretation. We point out that mechanical constraints can also explain those experimental recordings. In particular, the anatomy of the limb combined with the type of tasks studied and analysis used, suffice to give the appearance of neural synergies. To be clear, we do not disprove the neural synergy hypothesis. Rather, in the tradition of scientific debate, by showing an alternative explanation to the available data we challenge the community and ourselves to design novel experiments and analyses to conclusively test that hypothesis by ruling out the confounds we point out.

## Introduction

The muscle synergy hypothesis has received considerable attention in the neuroscience community (see [1] for a review). It posits that the central nervous system (CNS) activates muscles using the flexible combination of a small number of patterns. This hypothesis is commonly motivated as a potential mechanism by which the nervous system can simplify the control of a large number of muscles [2], [3], [4]. Counter-examples to the muscle synergy hypothesis have been observed for the control of hand musculature [5], [6]. We therefore set out to answer the question: is the human hand a unique system for not employing synergies, or are the muscle synergies detected in other neuromuscular systems actually of non-neural origin? Answering this question is crucial to making progress in the field of motor neuroscience.

The muscle synergy hypothesis has been notoriously difficult to prove or falsify [1]. Two distinct strategies have been employed to generate muscle activity to test this hypothesis: behavior in humans or animals, and direct stimulation of the motor system. The behavioral approach simply observes the electromyographic (EMG) activity in a large number of muscles during natural motor behavior, and uses computational techniques to identify consistent structure in the EMG signals across different tasks [3], [7], [8]. The stimulation approach artificially excites a variety of locations in the nervous system and shows that a relatively small number of muscle activation patterns emerge [9]. The behavioral approach has the advantage that it can be applied to a human or completely intact animal during natural behavior, but has the disadvantage that the task constraints could favor particular muscle activation patterns, independent of neural control [1]. The stimulation approach has the advantage that it is unaffected by the task constraints, but it is unclear whether the complete repertoire of muscle activation patterns can be elicited by these techniques [9]. Thus, existence of muscle synergies of neural origin has not been conclusively proven.

Muscle coordination studies using the behavioral approach are more relevant to natural human behavior [8] and disease states [7], and repeatedly show that muscle activations are constrained to a low-dimensional subspace across a variety of tasks. This potential evidence for the muscle synergy hypothesis comes from a number of behavioral studies, including cat postural control [3], [10], human postural control [11], [12], [13], human arm control [8], [14], human leg control [15], primate grasping [16], and natural lower limb behaviors of the frog [2]. The basis vectors of these low-dimensional subspaces are often called muscle synergies, and are taken to represent the underlying neural strategies to simplify multi-muscle control. An important class of behavioral experiments examining the muscle synergy hypothesis examines EMG responses to external perturbations (e.g. [3], [10], [12], [13], [14]). In this work, we show that such low dimensionality induced by external perturbation can be a product of unavoidable constraints related to movement. Another important class of behavioral experiments examines EMG during voluntary activation of muscles (e.g. [7], [8], [15]). In this work, we show that low EMG dimensionality during voluntary muscle force production could be related to task selection. Thus, we are fundamentally questioning the utility of the behavioral approach and the validity of its interpretation, because the synergies detected by these methods may not uniquely reflect neural strategies to simplify the control of multiple muscles.

## Methods

### Conceptual Motivation

**Figure 1:**
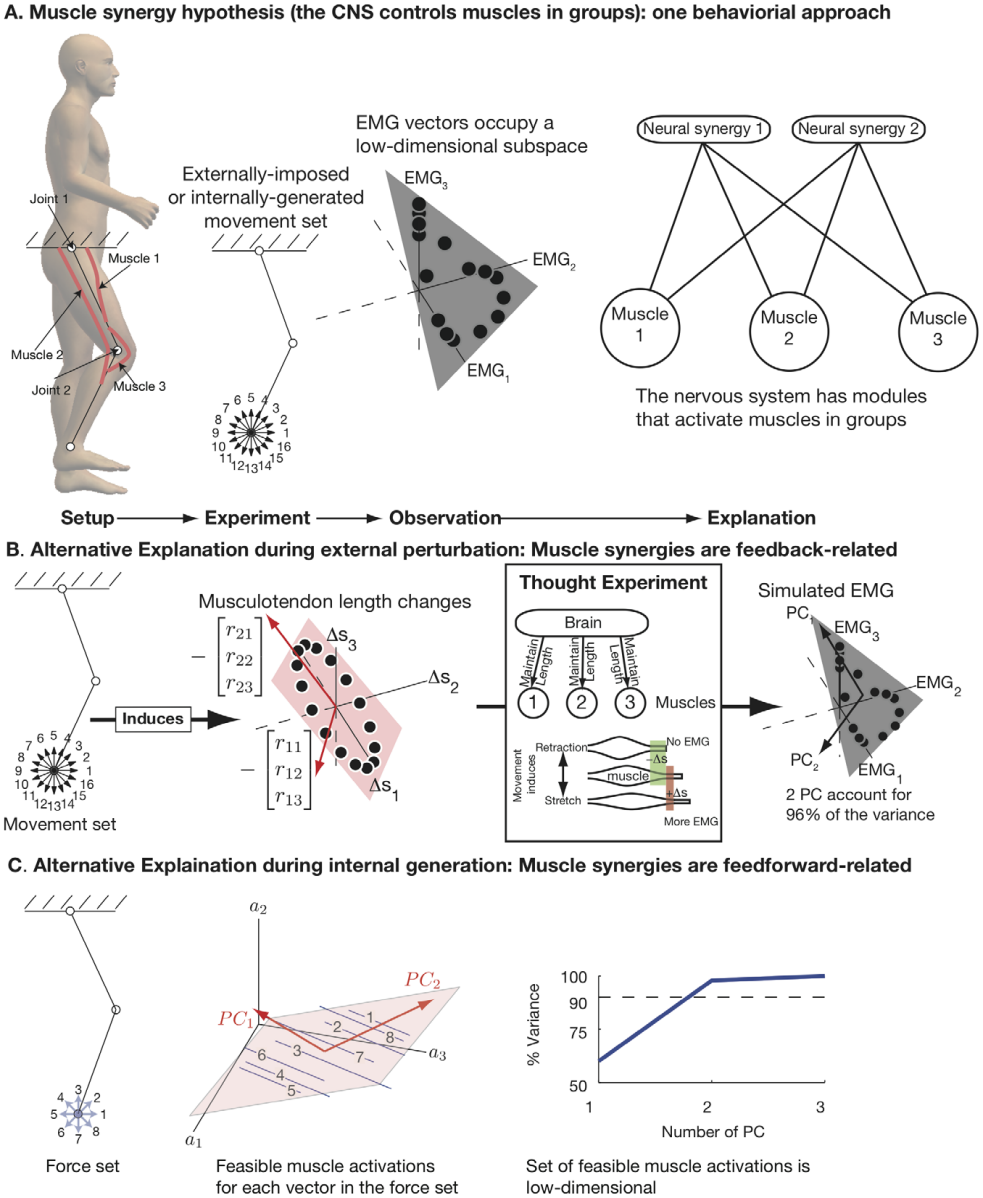
The nervous system does not need to control muscles in groups (muscle synergy hypothesis) to observe low-dimensional EMG. A. The behavioral approach to muscle synergies. Setup: A limb with more muscles than mechanical degrees-of-freedom (DOF). Experiment: the limb moves voluntarily (or is moved externally) in a large number of directions to span its workspace. Observation: The set of points in EMG space corresponding to each movement is in a low-dimensional subspace. Explanation: The nervous system has modules that activate muscles in groups to simply the control of movement. B. Alternative explanation 1: muscle synergies are movement related. The movement set induces a set of points in the space of musculotendon length change that is low dimensional (spanned by vectors composed of the muscle moment arms grouped by DOF). We perform a thought experiment by assuming that muscles are not controlled in groups by descending drive, but each muscle independently resists lengthening during small external perturbations of the endpoint. Only muscles lengthened by the perturbation will generate EMG; muscles shortened by the perturbation will not produce EMG. Using this thought experiment, we can generate simulated EMG, and we find that it is also low-dimensional. C. Alternative explanation 2: muscle synergies are feedforward-related. In this case, we imagine a limb producing forces in all directions at its endpoint. For each direction, the set of feasible muscle activations (assuming that each muscle can be activated between 0 and 1) can be calculated. These represent all the redundant activation vectors that will generate the same endpoint force. The set of all such feasible muscle activations across all directions is low-dimensional, as detected by PCA.

The behavioral approach to muscle synergies involves examining a limb controlled by multiple muscles (Figure 1A). The limb is moved, either voluntarily or externally, in a large number of directions in its workspace (Figure 1A). The set of EMG vectors, each of which describes the activity in multiple muscles for a particular direction of movement, is observed to be low-dimensional (Figure 1A). This observation is used to support the *muscle synergy hypothesis*, which posits that muscles can only be activated in groups (Figure 1A).

We begin by illustrating constraints that could appear as low-dimensional EMG without the muscle synergy hypothesis being true. We first do this graphically in simple system so it is clear that they apply generally and are not specific to any particular system or an artifact of a particular computational model (Figure 1). We then proceed to the detailed analysis of realistically complex neuromuscular systems. The 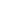
th element of moment arm matrix 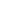, denoted 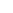, is the moment arm of the 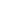
th muscle about the 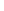
th joint. The angle of joint 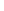 is denoted 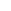, and the length of muscle 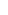
is denoted 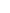. Any movement of this leg around a particular posture 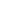 (Figure 1B) will induce changes in muscle length given by the equation 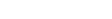. These unavoidable muscle length changes can be visualized as lying in a subspace (plane) spanned by two basis vectors, which are in fact the moment arms grouped by joint (Figure 1B).

To account for the causal interaction between musculotendon length changes and EMG during behavioral experiments with external perturbations, we perform a simple thought experiment. What pattern of EMG would we expect to see if there were no neural muscle synergies controlling muscles in groups, but each muscle independently resisted lengthening during the perturbation (Figure 1B)? This scenario would lead to increased EMG if the muscle were stretched, but no EMG if the movement induced the muscle to passively shorten, and would have the effect of stabilizing the reference posture. Examining the predicted EMG, we see that it would still be low-dimensional (Figure 1B), with 2 principal components accounting for 96% of the data variance. However, this low-dimensionality is not related to any neural controller designed to control muscles in groups (none was active). Rather, the low dimensionality arises naturally from biomechanical constraints and independent response of each muscle. We refer to these as feedback-related muscle synergies because they are mediated by afferent information. Notice that 2 synergies do not completely account for all of the simulated EMG variance, despite the fact that the external perturbations are 2-dimensional. This arises from the nonlinear relation between musculotendon length change and the resulting EMG.

Even if a task is internally driven and there is no external perturbation, the set of muscle activations will have a low dimensional structure even when the limb endpoint is driven in an exhaustive set of directions (Figure 1C shows the case of omnidirectional static force production). We refer to these as feedforward-related muscle synergies because the low dimensional structure of the muscle activations arises directly from the structure of the set of feasible motor commands. Because of muscle redundancy, a range of different muscle coordination patterns equivalently produce a same endpoint force vector (Figure 1C). The muscle coordination patterns that produce any single endpoint force vector are themselves a low-dimensional subset of muscle force space (in this case, a line that we accurately computed for this schematic model). But, perhaps counter-intuitively, even when all options for endpoint force in all directions are combined, the set of options available to the CNS is still low-dimensional (approximately spanned by only 2 principal components in this case). This is because the experiment, however exhaustive, still constrains the magnitude and direction of test forces. Therefore, the experimental design automatically constrains the observed combinations of muscle activity to a low-dimensional subspace, which could be misinterpreted as neurally-generated muscle synergies.

**Figure 2:**
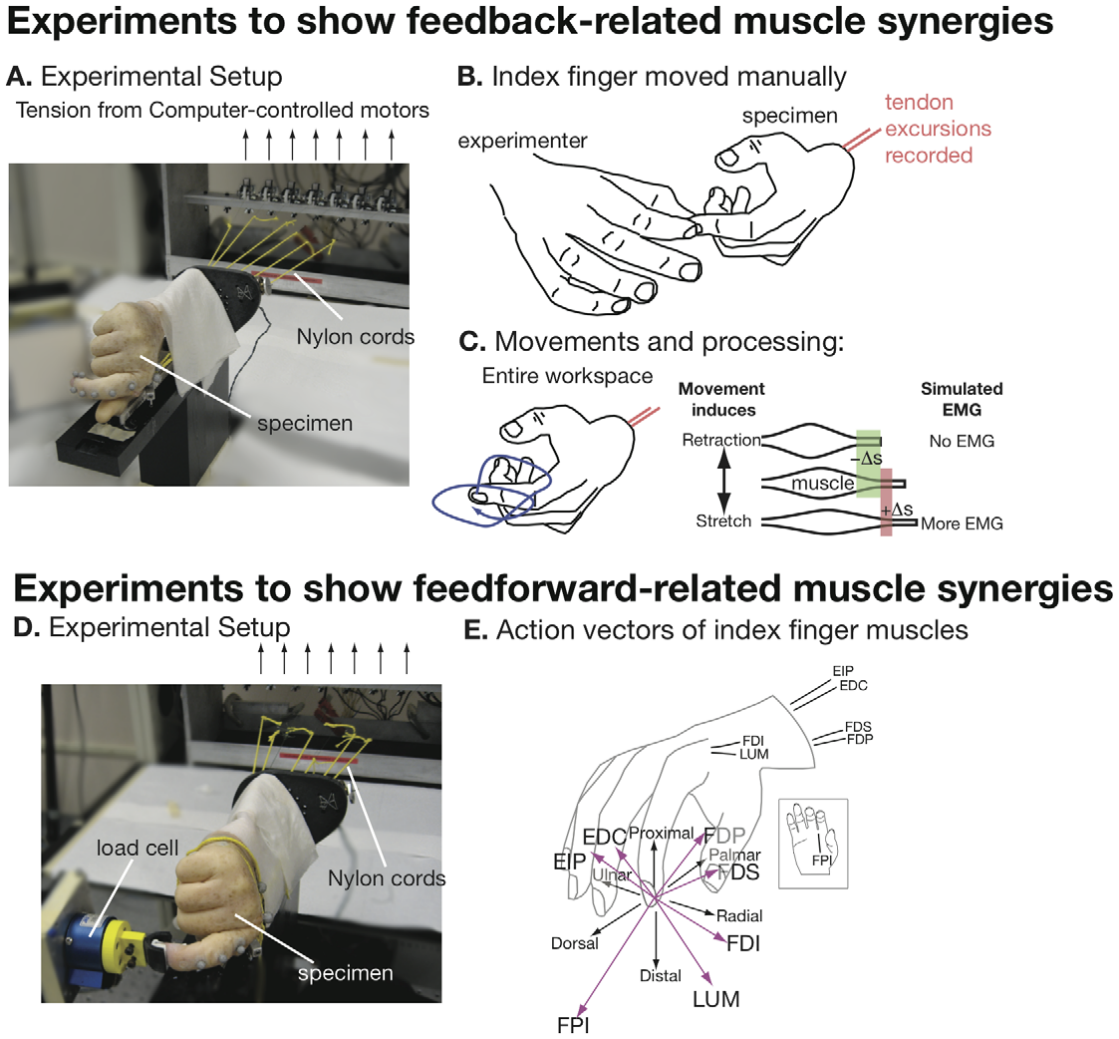
Experimental setup to demonstrate movement- and feedforward-related muscle synergies. A. To demonstrate feedback-related muscle synergies, we connected all seven index finger tendons in a cadaver specimen via Nylon cords to computer-controlled rotational motors, and left the index finger free. B. The experimenter forced the index finger to move while 5 N of tension was maintained on each tendon by a feedback controller. We simultaneously recorded the tendon excursions induced by each movement. C. We generated two-dimensional (entire workspace) movements by moving the finger randomly in its workspace around a starting posture. D. To demonstrate feedforward-related muscle synergies, we used the same setup as above, but rigidly coupled the fingertip to a 6-DOF load cell. We applied a sequence of muscle coordination patterns (see text) to determine the feasible forces that the finger could generate using its musculature. E. We performed linear regression on the fingertip load cell readings using the tendon tensions as factors, thus identifying the force vector in endpoint space caused by 1 N of tension in each tendon.

We found experimental evidence of feedback-related and feedforward-related muscle synergies in a cadaveric human hand (Figure 2) and evidence for them in a realistic model of the human leg, as described below.

### Experimental Methods

To demonstrate feedback-related and feedforward-related muscle synergies, we actuated the seven tendons of cadaveric index fingers with computer-controlled motors (Figure 2). As in prior work, we resected four fresh frozen cadaver arms at the mid-forearm level and dissected them to reveal the proximal end of the insertion tendons of all seven muscles controlling the index finger [17]: flexor digitorum profundus (FDP), flexor digitorum superficialis (FDS), extensor indicis (EI), extensor digitorum communis (EDC), first lumbrical (LUM), first dorsal interosseous (FDI), and first palmar interosseous (FPI). We fixed the specimen rigidly to a tabletop using an external fixator (Agee-WristJack, Hand Biomechanics Lab, Inc., Sacramento, CA), and we tied and glued the proximal tendons to Nylon cords attached to rotational motors. A real-time controller and custom-written software controlled the motors. Load cells measured the tension in each cord, which was fed back to the motor so that a desired amount of tension could be maintained on each tendon. A motion capture system (Vicon Motion Systems, Oxford, UK) recorded the angles of all index finger joints.

To demonstrate feedback-related muscle synergies the experimenter moved the finger in its workspace while changes in tendon length were recorded (Figure 2B). The motors actively maintained 5 N of tension on each tendon to prevent slackness. We generated movements of the fingertip at random until we filled the planar workspace of the finger around a starting posture (Figure 2C). We examined two postures, one with the index finger more extended and one with the index finger more flexed.

To demonstrate feedforward-related muscle synergies, we rigidly secured the index fingertip to a 6 DOF load cell (JR3, Woodland, CA) (Figure 2D)). A pre-programmed sequence of tension was then delivered to the tendons. An “active” tendon had 10 N applied to it, whereas an “inactive” tendon had 0 N applied to it. We delivered all possible activity combinations (for seven tendons there are 128 combinations: all possible combinations of 1 muscle active, 2 muscles active, 3 muscles active, …, all muscles active) in sequence to the specimen, holding each combination for 3 seconds and recording the average fingertip wrench (all forces and torques) exerted during this 3 second period. We then performed linear regression on the fingertip wrench using the applied tendon tension as the independent variable. This regression provided an action matrix 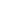 that predicted the fingertip wrench vector given the muscle activation vector (Figure 2E), and allowed us to quantify the goodness of that prediction. This action matrix included estimates for the maximum muscle force for the index finger muscles [17]. Analysis of the action matrix using computational geometry (see below) revealed all possible muscle coordination pattern options for endpoint force in all directions.

### Modeling Methods

We developed a model of the human leg to demonstrate that our results also apply to other parts of the motor system. To analyze feedback-related muscle synergies, we constructed a leg model using the 44 muscles and moment arms contained in a previously-validated lower extremity model [18]. We obtained the OpenSim implementation of this model from the Neuromuscular Models Library (simtk.org). For simplicity and without loss of generality, we only considered sagittal plane movement. We extracted the moment arms for the hip, knee, and ankle as a function of posture, and generated a 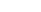 moment arm matrix 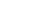. In addition, we extracted the estimated maximum isometric force for each muscle, and generated a 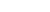 diagonal maximum force matrix 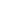. The Jacobian matrix was derived for a three-link planar manipulator [19], based on estimated anthropomorphic lengths for a 170 cm tall male [20].

We also adapted a well-accepted 14-muscle version [21] to analyze feedforward-related muscle synergies. We found that the 44-muscle model was of too high a dimensionality (44-D) for current computational geometry algorithms (see below), as complexity grows exponentially in the number of muscles. Fourteen muscles are on the same order as number of muscle recordings used to test for synergies, thus this model demonstrates the principles at work without loss of generality. The 14-D model contained 14 muscles/muscle groups (muscle/muscle group abbreviation in parentheses): medial and lateral gastrocnemius (gastroc), soleus (soleus), tibialis posterior (tibpost), peroneous brevis (perbrev), tibialis anterior (tibant), semimembranoseus/semitendenosis/biceps femoris long head (hamstring), biceps femoris short head (bfsh), rectus femoris (rectfem), gluteus medialis/glueteus minimus (glmed/min), adductor longus (addlong), iliacus (iliacus), tensor facia lata (tensfl), gluteus maximus (glmax). We obtained the maximal torque output for these muscle groups by averaging moment arms from the 44-muscle model, weighted by the estimated maximum isometric force. In so doing, the muscle groups have the same torque generating capabilities as the 44 individual muscles.

### Analysis Methods

#### Feedback-related muscle synergies

For the cadaveric experiments, we analyzed the measured musculotendon length changes by first converting them into a simulated EMG according to
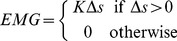
(1)which corresponds to the thought experiment described above (Figure 1B). 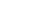, for each muscle, corresponded to the posture at the beginning of each trial. For the purposes of demonstrating feedback-related muscle synergies, we assumed that the feedback gain 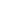 as the same for all muscles.

Using the computer model of the human leg, we estimated the musculotendon length changes that would be induced by relatively small (10 cm) movements of the foot in every direction. We held the orientation of the foot in space fixed. For 16 different directions 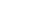, we constructed foot movements to be 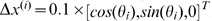. We then estimated the corresponding musculotendon length change in 44 dimensions according to 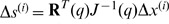. EMGs were simulated for each muscle and each direction of movement according to equation (1). Note that the Jacobian is invertible because the endpoint state includes the orientation of the ankle with respect to the ground [19]. This orientation was held fixed during the perturbations.

EMG dimensionality was assessed using principal components analysis (PCA) on the cloud of data points in muscle space generated by different movements. In addition, we tested the related hypothesis, suggested by some studies (e.g. [10]), that muscle synergy generalization (that is, a common set of muscle synergies can represent muscle activations in multiple postural conditions) may indicate the neural origin of such synergies. To do this, we used a PCA-based reconstruction procedure to determine if feedback-related muscle synergies would be expected to generalize across different postures. We first constructed a set of PCA basis vectors for the simulated EMG across movement direction in one posture. We then considered the leg model in a different posture. We determined the simulated EMG across movement direction in the second posture, and then determined how well these 44-dimensional EMG vectors could be reconstructed using the PCA basis vectors determined from the first posture. We quantified the goodness of reconstruction by calculating the variance accounted for (VAF) [10] value between the actual EMG vectors and those reconstructed from the PCA basis. Feedback-related synergy generalization was considered to occur when VAF>0.8 for all muscles [10].

#### Feedforward-related muscle synergies

To study feedforward-related muscle synergies we needed to describe all possible muscle coordination patterns that produce the same endpoint force vector. We did this using standard techniques in computational geometry [22], enumerating all vertices of the solution set in muscle activation space for given constraints on endpoint output [17], [23]. Target endpoint force vectors were chosen in 16 directions in the palmar-proximal plane of the index finger and the sagittal plane of the leg. We constrained additional force components to zero to keep the forces planar. We constrained the torque components to zero - which ensures that the endpoint forces could be applied statically to an object even if the endpoint were not rigidly constrained to that object. We performed PCA on the combination of the solution sets for all 16 directions of force output. We repeated this procedure to scan the entire solution space by increasing the force magnitude in increments (1 N for the finger and 50 N for the leg) until we could no longer find solutions in each direction.

## Results

### Feedback-Related Muscle Synergies

**Figure 3:**
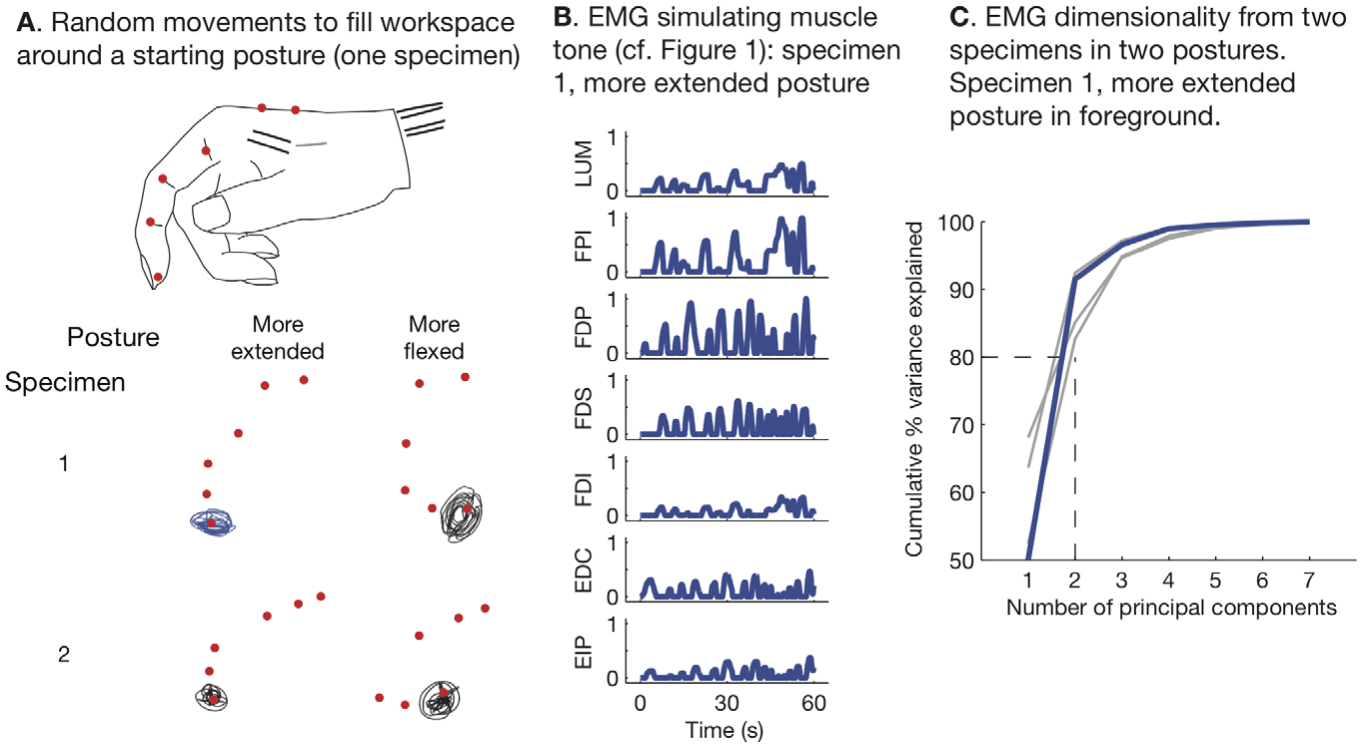
Feedback-related muscle synergies as raw data. A. The random movements that we produced with the fingertip are shown for both specimens and postures that we examined. The reference postures, from which excursions and simulated EMG are calculated, are shown by the circles. B. EMG is simulated using the thought experiment shown in Figure 1 across the time-series of data. The time series shown here corresponds to specimen 1 in the more extended posture. C. The simulated EMG was low-dimensional in all specimens and postures examined, with 2 principal components representing more than 80% of the simulated EMG variance in all cases.

We find that the dimension of the simulated EMG associated with feedback-related muscle synergies reflects the low dimensionality of the movement, creating the appearance of muscle synergies even in the absence of a specific neural controller (Figure 3). Movements of the fingertip had more than 80% of their variance explained by 2 principal components, and thus were largely confined to a plane (Figure 3A). These movements induced low-dimensional (synergistic) patterns of simulated EMG (Figure 3B) that mirrored the dimension of the movement (Figure 3C).

**Figure 4:**
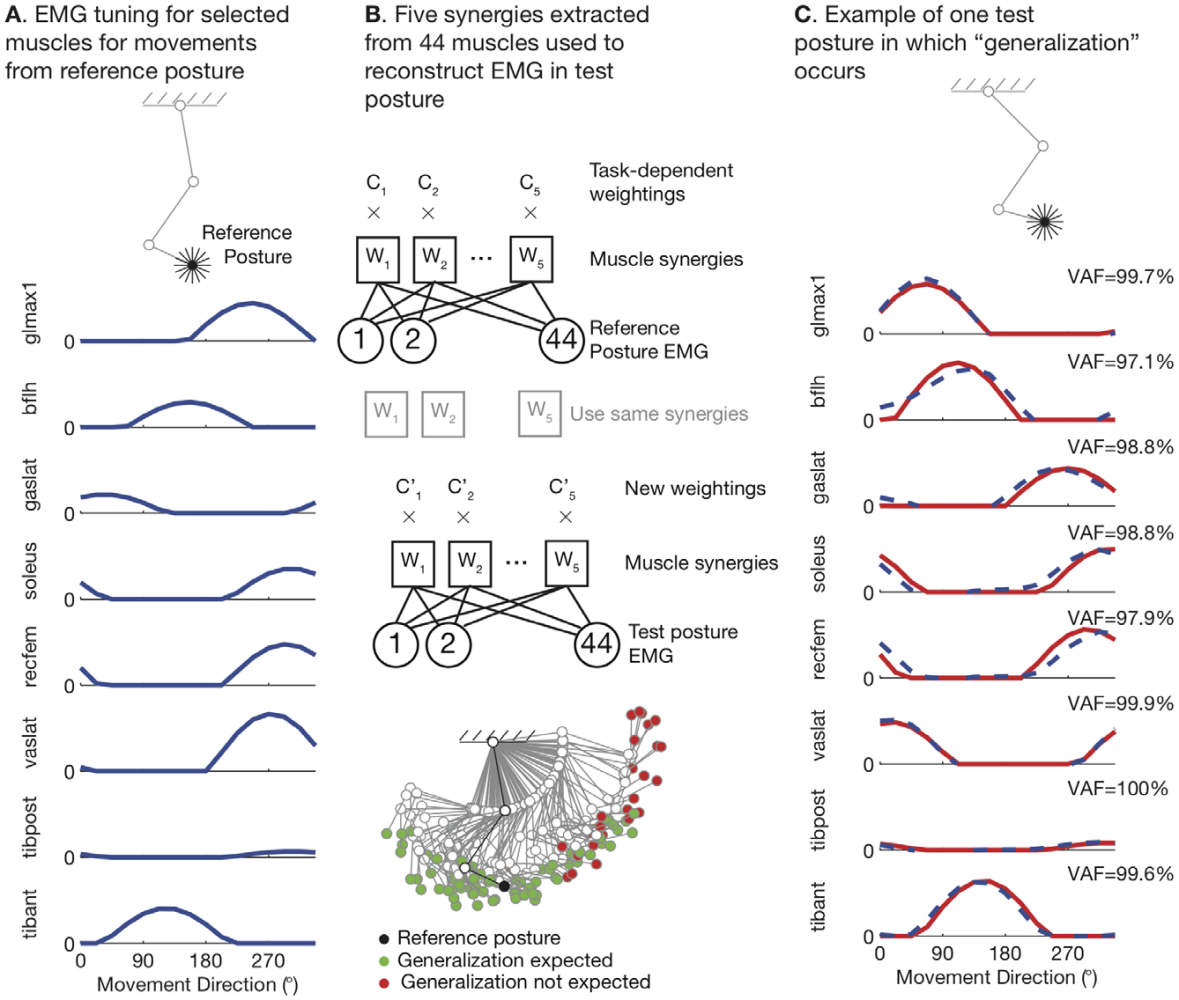
Feedback-related muscle synergies generalize across posture: human leg model. A. We simulated EMG for foot movements in 16 directions in a reference posture (only 8 of 44 muscles shown for clarity). B. We found that 80% of simulated EMG in the reference posture could be represented using only 5 PCA basis vectors (synergies). When we attempted to reconstruct simulated EMG from test postures scattered over the workspace using these 5 synergies, we found that generalization was expected over large portions of the workspace (more than 80% variance accounted for in each muscle). C. One example test posture is shown in which generalization would be expected to occur, without the muscle synergy hypothesis being true.

We tested if such feedback-related muscle synergies would exhibit features thought to support the neural-origin of muscle synergies. We chose to examine generalization across posture [10], meaning that the low-dimensional basis of muscle synergy vectors extracted from muscle activity in one posture could be used to reconstruct the muscle activity in a different posture. For our 44-muscle computational model of the human leg, 5 principal components were sufficient to explain more than 80% of the variance in simulated EMG across movements in 16 directions in a reference posture (Figure 4A). These 5 principal components were then used to reconstruct the simulated EMG from a different posture (test posture), in a test of synergy generalization (Figure 4B). A map of test postures where the reconstructed EMG accounted for more than 80% of the simulated EMG in the test posture indicates that synergy generalization is expected over a wide range of postures, without indicating that the muscle synergy hypothesis is true. An example of one test posture in which synergy generalization would be expected is shown (Figure 4C).

### Feedforward-Related Muscle Synergies

**Figure 5:**
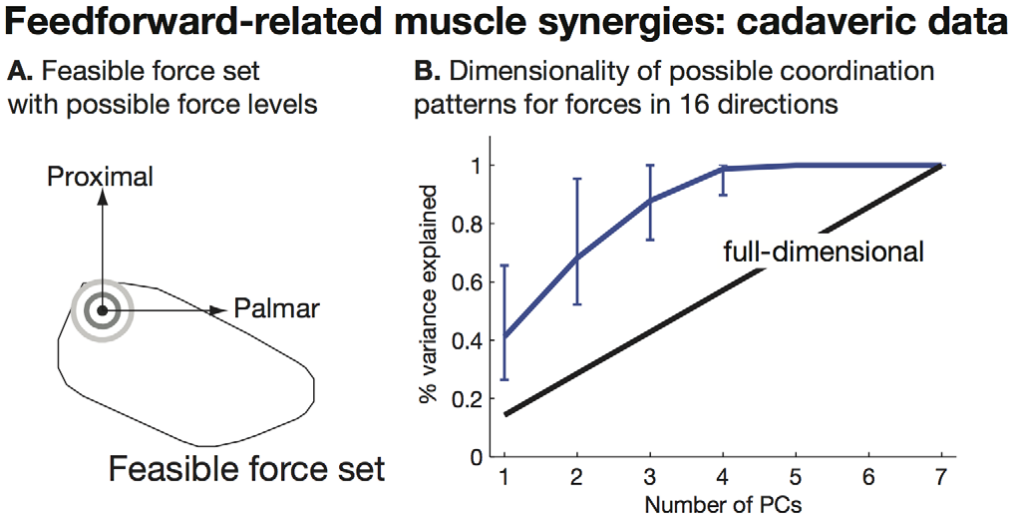
Feedforward-related muscle synergies: cadaveric data. A. We estimated the feasible force set in the palmar-proximal plane of the index fingertip for each specimen and posture examined (only one specimen/posture shown for clarity). We then determined how many force levels (concentric circles) could fit within the feasible force set. B. For each possible force level in each specimen at each posture, we estimated the dimensionality of the set of possible coordination patterns for force vectors in 16 directions. We found that the set of coordination patterns for omnidirectional tasks were low-dimensional regardless of the force level, specimen, or posture chosen (error bars indicate non-parametric 95% confidence intervals). This demonstrates that, even if the limb produces omnidirectional force, the mechanical nature of such experiments will always produce low-dimensional EMG data of the type that has been interpreted to reveal synergies of neural origin.

We demonstrate feedforward-related muscle synergies first on the cadaver index finger. We find, regardless of force magnitude (Figure 5A), that the set of muscle coordination patterns associated with fingertip forces in all directions is low-dimensional (Figure 5B). Thus, a low-dimensional muscle activation space should not be surprising in experiments with hand musculature, and does not by itself suggest a specific simplifying neural controller.

**Figure 6:**
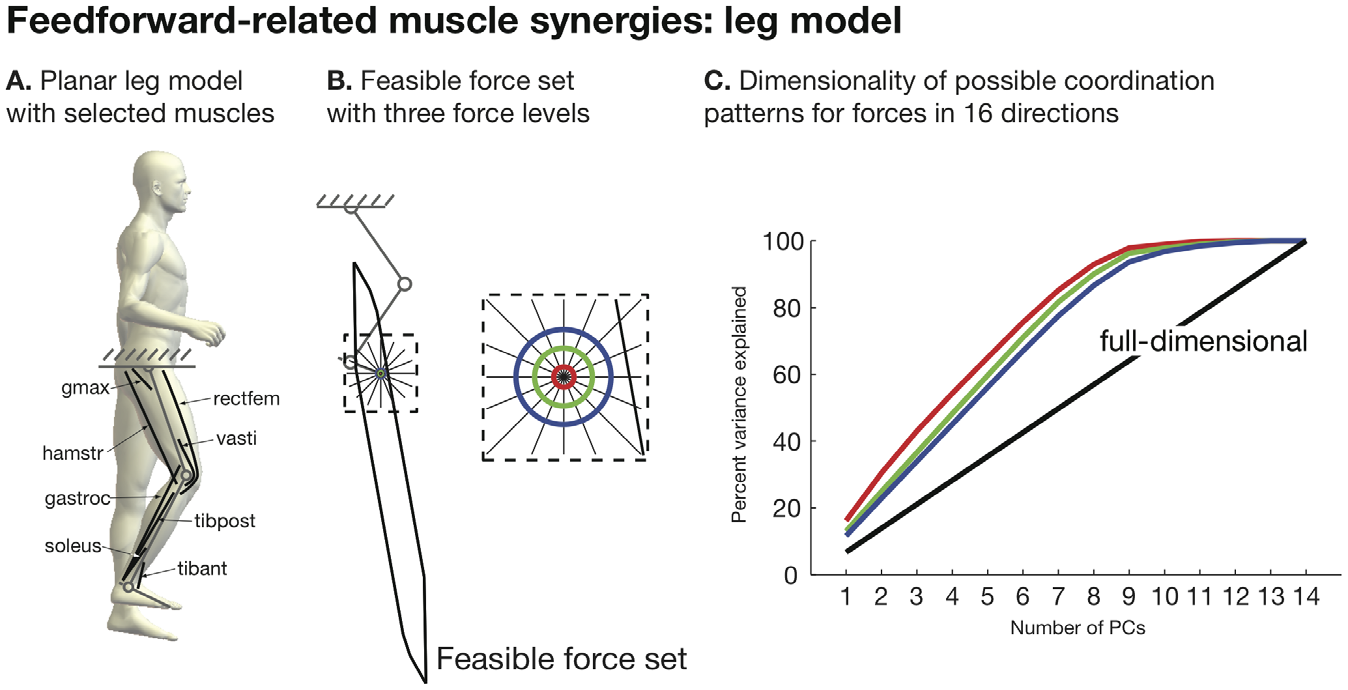
Feedforward-related muscle synergies: leg model. A. We used a simplified model with 14 muscles/muscle groups to make feasible the computation of all possible muscle coordination patterns for foot forces in different directions (8 muscles are illustrated for clarity). B. We found that the leg in this posture had a highly elongated feasible force set compatible with prior work [21], [36]. We found all force levels (concentric circles) that could fit within the feasible force set. C. We found that the set of possible coordination patterns for forces in 16 directions was low dimensional for all force magnitude levels.

We also demonstrate feedforward-related muscle synergies on our simplified 14-muscle computer model of the human leg (Figure 6A). Again, regardless of force magnitude (Figure 6B), the set of muscle coordination patterns associated with foot forces in all directions is low-dimensional (Figure 6C). However, it is clear from the rather large dimensionality of feedforward-related muscle synergies for the leg (7 synergies of 14 muscles are required to account for 80% of force variance) that the dimensionality of feedforward-related muscle synergies is not limited by the dimension of the task. Thus, while a low-dimensional muscle activation space should also not be surprising in experiments with leg musculature, and does not by itself suggest a specific neural controller, very low-dimensional EMG data during isometric force production would not be predicted by feedforward-related muscle synergies of the human leg. However, such predictions must be done using a biomechanical model on a experimental-specific basis for the limb being examined.

## Discussion

We performed this study to test whether non-neural constraints could produce the dimensionality reduction hypothesized to reflect neurally-established functional muscle groupings to relieve higher brain centers from controlling numerous muscles individually [1], [5], [6]. By providing clear counterexamples for limbs exerting endpoint static forces—or moving—in multiple directions, we demonstrate that two previously unrecognized non-neural constraints among muscles can also enforce such low-dimensionality, and thus give the appearance of muscle synergies. By confirming this alternative explanation to prior data and their interpretation, our work brings to light two fundamental non-neural constraints that need to be understood before muscle synergies of neural origin can be confidently disambiguated, found, and studied. We believe that properly controlling for these non-neural constraints is possible, and will enable studies that are capable of testing whether neural constraints are indeed present to reduce the dimensionality of the controller (i.e., the muscle synergy hypothesis). Thus, for example, it may be premature to attribute the emergence of such low dimensionality to neural sources [7] before other alternatives have been ruled out. To the best of our knowledge, the threshold for proving muscle synergies of neural origin exist has not been met because no study has adequately controlled for these non-neural interactions.

While other authors have previously used biomechanical models to explain muscle synergies [24], [25], to our knowledge no previous study has asked the more fundamental question of whether an experimenter could be led to conclude that the muscle synergy hypothesis was true when, in fact, constraints among EMG activity among muscles were coming from different sources.

Before discussing our results, we point out a potential source of confusion. If the nervous system is clearly generating the observed muscle activation patterns, how could one say that muscle synergies can be of non-neural origin? The key here is to distinguish between choices of motor commands the nervous system makes vs. constraints on the feasible motor commands the nervous can use [26]. If the nervous system could have used a large variety of different muscle coordination patterns for a given task, and only certain patterns are ever observed, then clearly some specific neural strategy selected the observed patterns. However, if only a given variety of muscle coordination patterns are feasible for a given task (due to, say, musculoskeletal geometry or the experimenter's selection of tasks), then clearly detecting a small variety of muscle coordination patterns does not suggest any specific neural strategy. Therefore, the goal of this study is to simply show by counterexample that non-neural interactions can also give rise to the dimensionality reduction thought to support the existence do muscle synergies of neural origin.

Along the feedback-related front, our study revealed a previously-unappreciated similarity between low-dimensional muscle synergies and a dimensionality reduction arising from coupling among muscle moment arms. In the muscle synergy hypothesis, this low-dimensional subspace is interpreted as a reflection of the CNS controlling the musculature with a small number of activation patterns. We show that such dimensionality reduction can also arise from mechanical coupling because there are far fewer joints than muscles. Our simulated EMG data came from a thought experiment that assumed that each muscle independently resists (and produced EMG) when a small externally imposed perturbation causes its lengthening [3], [10], [12], [13], [14]. This transformation between musculotendon length changes and EMG preserved the low-dimensionality of length changes and reflected them in the EMG signals. Of course, this is a simple transformation that may not apply to all experimental preparations. However, this transformation would produce low-dimensional EMG without the muscle synergy hypothesis being true, including generalization of EMG synergies across postures. It is likely that a wide variety of “well-behaved” transformations (e.g. monotonic) between length change and EMG will produce the same results found here. We found similar results for linear, exponential and sigmoidal relationships between muscle stretch and EMG. It is, therefore, the burden of the experimenter using the behavioral approach with external perturbations to demonstrate whether or not the actual transformation is causing EMG to reflect the low-dimensionality of induced muscultendon length changes. Feedback-related muscle synergies are strikingly similar to EMG synergies used in support of the muscle synergy hypothesis both in their low-dimensionality and in their generalization across posture [10], [12], [13].

Along the feedforward-related front, we have also demonstrated reasons to doubt available evidence for synergies of neural origin. *Feedforward-related muscle synergies* are inevitable in paradigms studying the generation of voluntary muscle force in redundant muscle systems. We show that, even in these presumably exhaustive explorations of force production, i.e. when endpoint forces of the same magnitude are generated in multiple directions, the mechanically defined set of muscle activation options available to the CNS is low-dimensional in the absence of any neural interactions. Feedforward-related muscle synergies in muscle activation space emerge, not from specific neural interactions, but from unconstrained variation in a task-irrelevant subspace. Thus, if experimentally-observed muscle synergies are feedforward-related, we would predict significant variation in muscle activation pattern among repeated trials of the same task. Such task-irrelevant inter-trial variability has been observed in muscle activation patterns [11], supporting the strong possibility that muscle synergies are feedforward-related, and do not reflect a specific dimensionality-reducing strategy employed by the CNS. Whereas we explored feedforward-related muscle synergies in the isometric context, coordination patterns would be similarly constrained during the production of voluntary feedforward movement because the equations of motion of the limb, combined with the desired trajectory in state space, define a manifold of feasible solutions for the control of movement [26], [27].

**Figure 7:**
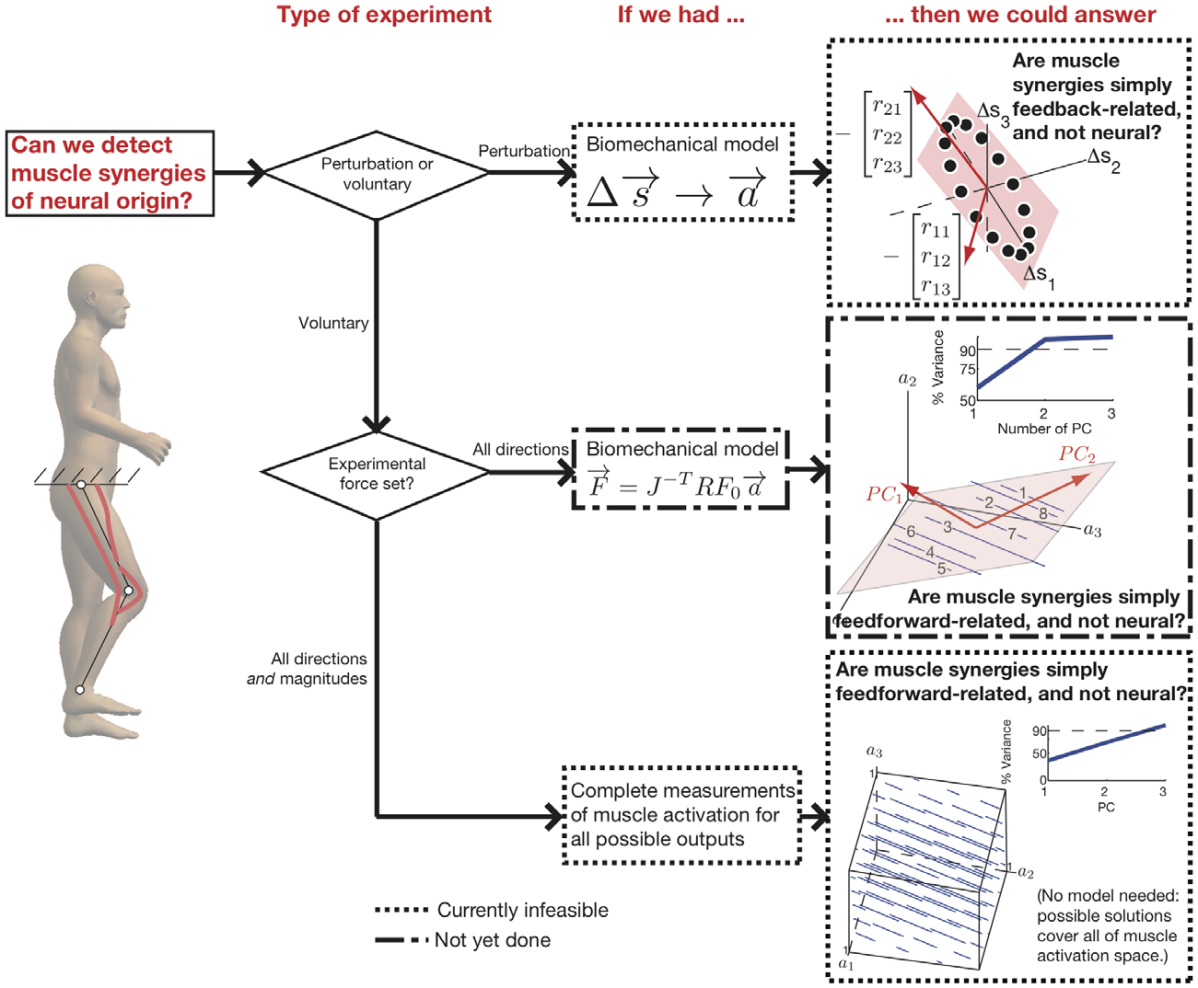
The question that we want to answer is “Can we find synergies of neural origin?” We believe that the first question to ask is whether the experimental paradigm is related to movement or force. The key is to disambiguate synergies of neural origin from potential confounds. If the experimental task primarily involved movement, then a biomechanical model must be proposed that relates musculotendon length changes to muscle activation. This model can then be used to predict whether the observed muscle synergies are feedback-related, and thus not neural in origin. If the experimental task primarily involved force, it is necessary to ask if the force set was only in all directions, or covered all possible directions *and* magnitudes. If only all force directions were covered at a fixed magnitude, then a biomechanical model must be proposed to predict endpoint force from muscle activation. It can then be determined if the observed muscle synergies are feedforward-related because the possible muscle coordination patterns occupy a low-dimensional space. Finally, if the experimental force set covered all directions and magnitudes (the entire feasible force set), and muscle synergies are observed, these synergies can be attributed to the nervous system without a biomechanical model. This is because the possible muscle coordination patterns become the entire full-dimensional muscle space once every possible endpoint force output has been visited.

We demonstrated feedforward-related muscle synergies using both cadaveric data and a simplified 14-muscle sagittal-plane computational model of the human leg. While it would have been ideal to analyze feedforward-related muscle synergies in the full 44-muscle leg model, vertex enumeration algorithms in computational geometry simply do not accommodate such large dimensionality. The simplified 14-muscle model suffices to provide the necessary counterexample to studies of muscle synergies for several reasons. It had many more muscles than endpoint degrees-of-freedom (14 muscles, 2 translational and 1 rotational DOFs at the endpoint). It had been previously validated and employed [21]. And 14 is on the order of the number of muscles that have been recorded from in studies of muscle synergies in the human leg ([12], [13] - 16 muscles). Thus we found that all muscle coordination patterns that produced endpoint force in all directions are embedded in a low-dimensional subspace of muscle activation space using three different approaches: a three-muscle schematic model (Figure 1), a 7-muscle model constructed directly from cadaveric data (Figure 6), and the 14-muscle leg model (Figure 7). Therefore, we conclude that feedforward-related muscle synergies are a general feature of neuromuscular systems producing forces in multiple directions, as demonstrated by three lines of evidence from computational and experimental data. We note that in the posture examined, analysis of this simplified leg model showed the least steep curve in variance explained vs. number of PC's. Nevertheless, these results support our conclusions because the aim of our work is demonstrate that there is real and unavoidable contribution of non-neural constraints on the low dimensionality of the neural command. In all cases this effect is real as the curves clearly depart from the unity line indicating no non-neural effects. So long as our examples show an effect of non-neural constraints, our point is made. An important, but often underestimated, issue in the literature is the subjective question of what constitutes a “significantly steep” slope that shows important neural or non-neural effects, and when does it begins to affect the interpretation of the data (for a detailed overview of estimation of degrees of freedom in motor systems and critical evaluation of PCA, please see [28]). Whether or not there is a significant non-neural effect is a question for researchers to decide depending on the goals of the study—and is beyond the scope of this work. Moreover, a motivation for our experimental work was to sidestep the tangential debate about whether or not any given leg or arm model is appropriately complex (see [29]). Our experiments to study force-related muscle synergies using actual cadaveric index fingers in multiple postures at their full natural complexity—where we know the ground truth of tendon actuation—reveal that as few as two synergies can account for more than 80% percent of force variance in seven muscles. This ratio of non-neural synergies that can explain c. 80% of the variance to number of muscles examined (2/7 = 0.29, see Figure 5B) is comparable to that attributed to synergies of neural origin in some studies, such as 5/19 = 0.26 in human reaching [8], 4/15 = 0.26 in cat postural control [3] and 6/16 = 0.38 in human postural control [12]. Therefore, our results provide direct counterexamples that strongly suggest that analyzing muscle synergies in the context of experiment-specific biomechanical models (Figure 7) is necessary to determine if feedforward-related muscle synergies could interfere with the interpretation of EMG data.

Can muscle synergies of neural origin ever be found? We use a flowchart to summarize how our findings suggest a new way forward to finding muscle synergies of neural origin (Figure 7). The key is to disambiguate synergies of neural origin from potential confounds. Our work reveals that feedback-related muscle synergies can be controlled for using a model that predicts musculotendon length changes on the basis of estimates of muscle activity, like EMG. We do not believe that such a model is currently feasible, largely because existing detailed mechanistic models of EMG apply to isometric contractions only [30] and often fail to realistically replicate fundamental features of EMG [31]. In contrast, current EMG techniques are a more reasonable estimate of activation level (and muscle force) than of musculotendon length change. The well-known limitations of EMG for these applications are discussed elsewhere [23], [26], [32], [33], but EMG nevertheless remains a reasonable and practical tool. Thus, our work reveals that force-induced muscle synergies could be controlled for in two ways. The first approach would be an experimental paradigm which could proceed with an incomplete sampling of the feasible force output of the limb. For example, the experiment could employ endpoint outputs in every direction as is currently used, but it would be coupled with an experiment-specific computational model to estimate the features of muscle activation that are not explained by the constraints of the task. Such a computational model could predict the possible coordination patterns, and determine if muscle synergies were observed simply because the possible coordination patterns were low-dimensional. This approach has not yet been taken to study muscle synergies, but we believe it to be the most productive way forward. A prior studies has approximated such approach [6], [34], and their conclusions did not support the presence of synergies of neural origin. The second approach would be an experimental paradigm that requires the limb to generate every possible endpoint output (in magnitude and direction). If dimensionality reduction in muscle activation space were observed, it could then be unambiguously interpreted as muscle synergies of neural origin. While the feasible force set has been estimated in human subjects [35], such experiments may prove prohibitively long or fatiguing while maintaining intramuscular electrodes in position to reliably record from all muscles of the limb or finger.

Disambiguating muscle synergies of neural origin from those of non-neural origin is essential not only for basic research in motor neuroscience, but also for clinical populations. Muscle synergy structure has been reported to be similar between the affected and unaffected arms of stroke survivors [7]. Since the biomechanical structure of these limbs may be very similar, non-neural feedback-related and feedforward-related muscle synergies could have the primary contributors to this finding. Thus, controlling for these non-neural interactions may be essential to designing the most effective rehabilitation strategies.
